# Draft Genome Sequences of Obligate Methylotrophs Methylovorus sp. Strain MM2 and Methylobacillus sp. Strain MM3, Isolated from Grassland Soil

**DOI:** 10.1128/MRA.00824-18

**Published:** 2018-08-30

**Authors:** Michael Christopher Macey, Jennifer Pratscher, Andrew Crombie, J. Colin Murrell

**Affiliations:** a School of Environmental Sciences, University of East Anglia, Norwich, United Kingdom; b School of Biological Sciences, University of East Anglia, Norwich, United Kingdom; Loyola University Chicago

## Abstract

Methylotrophs of the family Methylophilaceae were isolated from grassland soil. Here, we report the draft genome sequences of two obligate methylotrophs, Methylovorus sp. strain MM2 and Methylobacillus sp. strain MM3. These genome sequences provide further insights into the genetic and metabolic diversity of the Methylophilaceae.

## Abstract (precis)

Methylotrophs of the family Methylophilaceae were isolated from grassland soil. Here, we report the draft genome sequences of two obligate methylotrophs, Methylovorus sp.

## ANNOUNCEMENT

The family Methylophilaceae is composed of four genera containing facultative and obligate methanol-utilizing methylotrophs (1–4). Members of the Methylophilaceae have been isolated from a range of locations, including terrestrial and marine environments (5–10). Here, we report the draft genome sequences of two obligate methylotrophs, Methylovorus sp. strain MM2 and Methylobacillus sp. strain MM3. The obligate nature of these methylotrophs was confirmed through growth experiments. The strains were isolated from soil collected at a 5-cm depth from grassland in Bawburgh, Norfolk, United Kingdom (52.6276 N, 1.1784 E).

Genome sequencing was performed by MicrobesNG using the Illumina MiSeq platform, producing 2 × 250-bp paired-end reads. Trimmed sequences were assembled using SPAdes version 3.7.1, and genome annotation was performed using the RAST annotation server (http://rast.nmpdr.org) (11, 12). Coverage of the genomes was calculated using BWA, SAMtools, and BEDTools genomecov (13–15). The Methylovorus sp. MM2 genome is composed of 27 contigs and includes 2,291 coding sequences (CDSs), 1 16S rRNA gene copy, and 46 tRNAs. The genome size is 2.42 Mb, with 46% G+C content. The genome of Methylobacillus sp. MM3, with 2.95 Mb and 57% G+C content, is composed of 64 contigs and includes 2,897 CDSs and 3 copies of 16S rRNA genes. Both genomes had 30-fold coverage.

Both genomes contain pyrroloquinoline quinone methanol dehydrogenases. Methylobacillus sp. MM3 possesses three separate gene clusters for the alternative methanol dehydrogenase XoxF (16, 17) and no copies of the canonical methanol dehydrogenase-encoding genes *mxaFI*. Methylovorus sp. MM2 possesses three copies of *xoxF* and one set of the genes *mxaFI*. All genes encoding the *N*-methylglutamate pathway for methylamine utilization (*mgdABC, gmaS*, and *mgsABC*) (18, 19) are present only in the genome of Methylobacillus sp. MM3, in addition to genes that encode dimethylamine dehydrogenase and trimethylamine dehydrogenase enzymes (*dmd* and *tmd*) (20, 21). The genes for an assimilatory nitrate reductase (*nasAB*) and the complete denitrification pathway (*narGHI, nirK, nirS, norB,* and *nosZ*) are present in the genome of Methylobacillus sp. MM3, while Methylovorus sp. MM2 possesses only an assimilatory nitrate reductase (*nasAB*) and a dissimilatory nitrite reductase (*nirBD*).

### Data availability.

These whole-genome shotgun projects have been deposited at DDBJ/ENA/GenBank under accession numbers LXTQ00000000 for Methylobacillus sp. MM3 and LXUF00000000 for Methylovorus sp. MM2. The versions described in this paper are the first versions. The strains are available from the authors upon request.

## References

[1] Kaparullina EN, Trotsenko YA, Doronina NV 2017 *Methylobacillus methanolivorans* sp. nov., a novel non-pigmented obligately methylotrophic bacterium. Int J Syst Evol Microbiol 67:425–431. doi:10.1099/ijsem.0.001646. 27902271

[2] Doronina NV, Kaparullina EN, Trotsenko YA 2016 Emended description of *Methylovorus glucosotrophus* Govorukhina and Trotsenko 1991. Microbiology 85:548–552. doi:10.1134/S0026261716050040. 29364598

[3] Kalyuzhnaya MG, Beck DAC, Vorobev A, Smalley N, Kunkel DD, Lidstrom ME, Chistoserdova L 2012 Novel methylotrophic isolates from lake sediment, description of *Methylotenera versatilis* sp. nov. and emended description of the genus *Methylotenera*. Int J Syst Evol Microbiol 62:106–111. doi:10.1099/ijs.0.029165-0. 21335496

[4] Jenkins O, Byrom D, Jones D 1987 *Methylophilus*: a new genus of methanol-utilizing bacteria. Int J Syst Evol Microbiol 37:446–448. doi:10.1099/00207713-37-4-446.

[5] Doronina NV, Gogleva AA, Trotsenko YA 2012 *Methylophilus glucosoxydans* sp. nov., a restricted facultative methylotroph from rice rhizosphere. Int J Syst Evol Microbiol 62:196–201. doi:10.1099/ijs.0.024620-0. 21378135

[6] Gogleva AA, Kaparullina EN, Doronina NV, Trotsenko YA 2011 *Methylobacillus arboreus* sp. nov., and *Methylobacillus gramineus* sp. nov., novel non-pigmented obligately methylotrophic bacteria associated with plants. Syst Appl Microbiol 34:477–481. doi:10.1016/j.syapm.2011.03.005. 21640537

[7] Doronina NV, Trotsenko YA, Kolganova TV, Tourova TP, Salkinoja-Salonen MS 2004 *Methylobacillus pratensis* sp. nov., a novel non-pigmented, aerobic, obligately methylotrophic bacterium isolated from meadow grass. Int J Syst Evol Microbiol 54:1453–1457. doi:10.1099/ijs.0.02956-0. 15388694

[8] Madhaiyan M, Poonguzhali S, Kwon S-W, Sa T-M 2009 *Methylophilus rhizosphaerae* sp. nov., a restricted facultative methylotroph isolated from rice rhizosphere soil. Int J Syst Evol Microbiol 59:2904–2908. doi:10.1099/ijs.0.009811-0. 19628595

[9] Doronina NV, Kaparullina EN, Trotsenko YA 2011 *Methylovorus menthalis*, a novel species of aerobic obligate methylobacteria associated with plants. Microbiology 80:713–719. doi:10.1134/S0026261711050043. 22168014

[10] Xia F, Zou B, Shen C, Zhu T, Gao X-H, Quan Z-X 2015 Complete genome sequence of *Methylophilus* sp. TWE2 isolated from methane oxidation enrichment culture of tap-water. J Biotechnol 211:121–122. doi:10.1016/j.jbiotec.2015.07.023. 26253961

[11] Bankevich A, Nurk S, Antipov D, Gurevich AA, Dvorkin M, Kulikov AS, Lesin VM, Nikolenko SI, Pham S, Prjibelski AD, Pyshkin AV, Sirotkin AV, Vyahhi N, Tesler G, Alekseyev MA, Pevzner PA 2012 SPAdes: a new genome assembly algorithm and its applications to single-cell sequencing. J Comput Biol 19:455–477. doi:10.1089/cmb.2012.0021. 22506599 PMC3342519

[12] Aziz RK, Bartels D, Best AA, Dejongh M, Disz T, Edwards RA, Formsma K, Gerdes S, Glass EM, Kubal M, Meyer F, Olsen GJ, Olson R, Osterman AL, Overbeek RA, Mcneil LK, Paarmann D, Paczian T, Parrello B, Pusch GD, Reich C, Stevens R, Vassieva O, Vonstein V, Wilke A, Zagnitko O 2008 The RAST server: Rapid Annotations using Subsystems Technology. Nucleic Acids Res 9:75. doi:10.1186/1471-2164-9-75. PMC2265698 18261238

[13] Li H, Durbin R 2009 Fast and accurate short read alignment with Burrows-Wheeler transform. Bioinformatics 25:1754–1760. doi:10.1093/bioinformatics/btp324. 19451168 PMC2705234

[14] Li H, Handsaker B, Wysoker A, Fennell T, Ruan J, Homer N, Marth G, Abecasis G, Durbin R, 1000 Genome Project Data Processing Subgroup 2009 The Sequence Alignment/Map (SAM) format and SAMtools. Bioinformatics 25:2078–2079. doi:10.1093/bioinformatics/btp352. 19505943 PMC2723002

[15] Quinlan AR, Hall IM 2010 BEDTools: a flexible suite of utilities for comparing genomic features. Bioinformatics 26:841–842. doi:10.1093/bioinformatics/btq033. 20110278 PMC2832824

[16] Chistoserdova L 2011 Modularity of methylotrophy, revisited. Environ Microbiol 13:2603–2622. doi:10.1111/j.1462-2920.2011.02464.x. 21443740

[17] Keltjens JT, Pol A, Reimann J, Op den Camp HJM 2014 PQQ-dependent methanol dehydrogenases: rare-earth elements make a difference. Appl Microbiol Biotechnol 98:6163–6183. doi:10.1007/s00253-014-5766-8. 24816778

[18] Chen Y, Scanlan J, Song L, Crombie A, Rahman MT, Schafer H, Murrell JC 2010 γ-Glutamylmethylamide is an essential intermediate in the metabolism of methylamine by *Methylocella silvestris*. Appl Environ Microbiol 76:4530–4537. doi:10.1128/AEM.00739-10. 20472738 PMC2897447

[19] Latypova E, Yang S, Wang Y-S, Wang T, Chavkin TA, Hackett M, Schäfer H, Kalyuzhnaya MG 2010 Genetics of the glutamate-mediated methylamine utilization pathway in the facultative methylotrophic beta-proteobacterium *Methyloversatilis universalis* FAM5. Mol Microbiol 75:426–439. doi:10.1111/j.1365-2958.2009.06989.x. 19943898

[20] Boyd G, Math FS, Leonard C, Scrutton NS 1992 Trimethylamine dehydrogenase of bacterium W_3_A_1_ molecular cloning, sequence determination and over-expression of the gene. FEBS Lett 308:271–276. doi:10.1016/0014-5793(92)81291-S. 1505666

[21] Yang C-C, Packman LC, Scrutton NS 1995 The primary structure of *Hyphomicrobium* X dimethylamine dehydrogenase. Eur J Biochem 232:264–271. doi:10.1111/j.1432-1033.1995.tb20808.x. 7556160

